# Weakly nonlinear propagation of focused ultrasound in bubbly liquids with a thermal effect: Derivation of two cases of Khokolov–Zabolotskaya–Kuznetsoz equations

**DOI:** 10.1016/j.ultsonch.2022.105911

**Published:** 2022-01-11

**Authors:** Shunsuke Kagami, Tetsuya Kanagawa

**Affiliations:** a Department of Engineering Mechanics and Energy, Graduate School of Systems and Information Engineering, University of Tsukuba, 1-1-1 Tennodai, Tsukuba 305-8573, Japan; b Department of Engineering Mechanics and Energy, Faculty of Engineering, Information and Systems, University of Tsukuba, 1-1-1 Tennodai, Tsukuba 305-8573, Japan

**Keywords:** Bubble dynamics, Bubbly liquid, Focused ultrasound, Weakly nonlinear wave, Khokhlov–Zabolotskaya–Kuznetsov equation, High-intensity focused ultrasound

## Abstract

A physico-mathematical model composed of a single equation that consistently describes nonlinear focused ultrasound, bubble oscillations, and temperature fluctuations is theoretically proposed for microbubble-enhanced medical applications. The Khokhlov–Zabolotskaya–Kuznetsov (KZK) equation that has been widely used as a simplified model for nonlinear propagation of focused ultrasound in pure liquid is extended to that in liquid containing many spherical microbubbles, by applying the method of multiple scales to the volumetric averaged basic equations for bubbly liquids. As a result, for two-dimensional and three-dimensional cases, KZK equations composed of the linear combination of nonlinear, dissipation, dispersion, and focusing terms are derived. Especially, the dissipation term depends on three factors, i.e., interfacial liquid viscosity, liquid compressibility, and thermal conductivity of gas inside bubbles; the thermal conduction is evaluated by using four types of temperature gradient models. Finally, we numerically solve the derived KZK equation and show a moderate temperature rise appropriate to medical applications.

## Highlights

• Derivation of physico-mathematical simplified model for nonlinear focused ultrasound in liquids containing many bubbles.
• Consistent incorporation of ultrasound propagation, bubble oscillation, and temperature fluctuation into single equation.
• Spatially two- and three-dimensional cases.
• Moderate temperature rise via a numerical analysis.

## 1 Introduction

Focused ultrasound is widely used in medical applications for both diagnosis and treatment of various conditions [1]. Ultrasound imaging [2] is a diagnosis method that can be performed in real time. High-intensity focused ultrasound (HIFU) treatment [3], [4], [5] is a low-invasive treatment method that is utilized for tumor ablation therapy and shock wave lithotripsy (SWL). Both methods do not present a risk of radiation.

The Khokhlov–Zabolotskaya–Kuznetsov (KZK) equation [6], [7], describing a weakly nonlinear propagation of focused ultrasound waves in pure liquid, has been widely used as a physico-mathematical model for medical applications [8], [9], [10], [11], [12], [13], [14], [15], [16], [17], [18], [19], [20], [21], [22], [23], [24], [25], [26], [27], [28], [29], [30], [31], [32], [33], [34], [35], [36], [37], [38], [39], [40], [41]. For SWL treatments, the calculation of pressure distributions using the KZK equation has been found to be in agreement with experimental results [9], [10]. For tumor ablation therapies, the temperature distribution must be calculated, but the existing KZK equation is written in a form describing the pressure distribution. Therefore, the temperature distribution is calculated [11], [12], [13], [14], [15], [16], [17], [18], [19], [20], [21] by combining the KZK and bio–heat equations [42], which has been found to agree with experimental results [11], [12], [13], [14], [15], [16], [17], [18], [19]. Moreover, some studies [11], [12], [13], [14], [15] have compared numerical results between the nonlinear (KZK equation) and linear models, reporting that the consideration of nonlinearity increases the accuracy of the numerical result. The KZK equation can be divided into two types as a quasi-plane wave: the two-dimensional (2D) and three-dimensional (3D) spatial forms (see also Section 2). The 2D KZK equation has been used [1], [9], [10], [11], [12], [13], [14], [15], [16], [17], [18], [20], [21], [22], [23], [24], [25], [26], [27], [28], [29], [30], [31] for axisymmetric propagation of focused ultrasound and can be calculated at a lower computational cost. However, for describing the nonuniformity of the medium [19], [32], [33], [34] and the ultrasound radiated by a rectangular sound source [33], [34], [35], [36], [37], [38], [39], which are important for ultrasound imaging, the 3D KZK equation is required and has been used effectively in several studies [19], [32], [33], [34], [35], [36], [37], [38], [39], [40], [41].

In recent reports, the use of bubbles has been shown to improve the efficiency of medical applications. For ultrasound imaging, microbubbles can be used as ultrasound contrast agents to significantly improve the resolution of images [43], [44], [45]. For tumor ablation therapy by HIFU, thermal effects radiated by oscillating bubbles can improve the efficiency of temperature rise [46], [47], [48], [49], [50], [51]. Kaneko et al. [48] carried out an experiment using rabbit liver and reported that the use of bubbles approximately doubled the ablated volume and increased the temperature rise by approximately 7 °C during 60 s of HIFU radiation. Chang et al. [51] carried out an experiment using a tissue-mimicking phantom and reported that the use of bubbles increased the ablated volume approximately sixfold during 60 s of HIFU radiation.

A physico-mathematical model for medical applications utilizing bubbles should describe the nonlinearity of both ultrasound propagation and bubble oscillation. Vanhille [30] focused on a medium containing a bubble cloud only in the pre-focal zone and proposed a method of solving the KZK equation for the area without bubbles and solving the linear wave equation and bubble dynamics equation (Rayleigh–Plesset equation) separately for the area near the bubble clouds. However, for the area near the bubble clouds, the nonlinearity of bubble oscillation was considered but that of ultrasound propagation was not. Hence, extending the KZK equation from pure liquid to liquid containing bubbles can reduce the computational cost and consistently describe the nonlinear effects of both ultrasound propagation and bubble oscillation. Khismatullin and Akhatov [52] derived a physico-mathematical model similar to the KZK equation for a liquid containing many bubbles, but dissipation effects were not considered (i.e., the Kadomtsev–Petviashvili equation). Our previous study [53], [54] was the first to succeed in deriving a generalized KZK equation (i.e., the KZK equation including a dispersion term as third-order derivative due to bubble oscillations) with dissipation effects by the viscosity at bubble–liquid interface and the compressibility of the liquid phase, based on the basic equations for a liquid containing many spherical bubbles. However, temperature fluctuation and thermal conduction of the gas inside the bubbles was not accounted for. In some cases of tumor ablation therapy by HIFU, the temperature of the area near the focus can rise by over 80 °C [5], making a description of temperature fluctuation and thermal effects necessary.

Recently, we proposed another generalized KZK equation describing the temperature fluctuation and thermal conduction of the gas inside bubbles [55], [56] by introducing the energy equation for the gas inside bubbles [57]. However, this study [55], [56] has some theoretical limitations: (i) Only the 2D KZK equation was derived because axisymmetric propagation was assumed. (ii) Volumetric averaged equations based on a mixture model [58] for bubbly liquids were used. However, it is reported [59] that a two-fluid model [60] can describe the dependence of the initial condition of the medium more accurately than the mixture model. (iii) The temperature gradient term of the energy equation [57] needs to be rewritten. Although many temperature gradient models have been proposed [61], [62], [63], [64], only one model [62] was used in that study [55], [56], and hence there is no knowledge of which model is the most accurate for modelling medical applications. In addition to these points, numerical solutions of the KZK equation for bubbly liquids have yet to be obtained.

The purpose of this study is the derivation of 2D and 3D generalized KZK equations for bubbly liquids using the volumetric averaged equations based on the two-fluid model [60], with a numerical example of the newly obtained equations provided. This paper is organized as follows: Section 2 introduces the basic equations; including the volumetric averaged equations based on a two-fluid model [60], the energy equation for the gas inside a bubble [57], and the temperature gradient models [61], [62], [63], [64]. The perturbation expansions based on the multiple-scales method are demonstrated [65]. Section 3 presents the method for the derivation of the 2D and 3D KZK equations. The resultant 2D and 3D KZK equations are represented as the linear combination of nonlinear, dissipation, dispersion, and diffraction terms. The dissipation term is divided into three factors; the interfacial viscosity, liquid compressibility, and thermal conductivity of gas. Section 4 includes a numerical example of the newly obtained equations for the spatial distribution of temperature fluctuations. The finite-difference time-domain (FDTD) method developed by Lee and Hamilton [22], [23] that is widely used as a numerical method for solving the KZK equation for pure liquids is extended to bubbly liquids in this study. The time development of the dissipation term is shown for the interfacial viscosity, liquid compressibility, and thermal conductivity of the gas. The calculation is carried out for three types of gas inside bubbles: Argon, air, and sulfur hexafluoride (${\mathrm{SF}}_{6}$). The results show that Argon and ${\mathrm{SF}}_{6}$ gas are most effective for HIFU treatment and ultrasound imaging, respectively.

## 2 Formulation of the problem

### 2.1 Problem statement

**Fig. 1:**
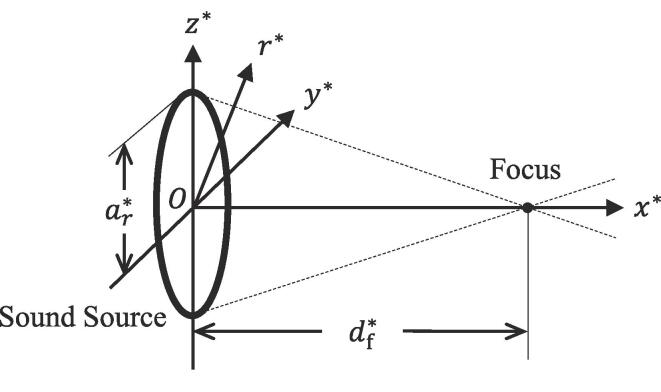
Schematic of model. Propagation of focused ultrasound in case of circular sound source.

Weakly nonlinear propagation of focused ultrasound in a compressible liquid containing many spherical bubbles is considered in this study. The sound beam was radiated from a sound source in the bubbly liquid (Fig. 1). The surface shape of the sound source is not restricted to planar form, as it may be concave or convex; the former yields a focused beam and the latter a spreading beam. As shown in Fig. 1, the origin is set at the center of the sound source, the $x^{*}$ axis is normal to the surface of the sound source, the $r^{*}$, $y^{*}$, and $z^{*}$ axes are the distances from the $x^{*}$ axis. This provides the equation of

(1) $$r^{*}=\sqrt{y^{*2}+z^{*2}}\text{.}$$

The 2D and 3D KZK equations are derived by assuming the quasi-plane waves, i.e., weakly diffracted (weakly focused or weakly spreading) waves. As the assumption of quasi-plane waves for the derivation of 2D KZK equation, we assume that the typical wavelength $L^{*}$ is significantly longer than the initial bubble radius $R_{0}^{*}$, and the radius of the circular sound source $a_{r}^{*}$ is significantly longer than $L^{*}$, as in our previous work [53], [54], [55], [56].

(2) $$R_{0}^{*}\ll L^{*}\ll a_{r}^{*}\text{.}$$

Although the diameter of the sound source was used in our previous work [53], [54], [55], [56], the radius $a_{r}^{*}$ is used in this study for applicability to the numerical calculation, but this does not significantly affect the result [53], [54], [55], [56]. In this study, we extend the theory to the 3D KZK equation, where the profile of the sound source is not restricted to a circular form and may be elliptical or rectangular, hence, we introduce the assumption of

(3) $$R_{0}^{*}\ll L^{*}\ll a_{y}^{*}\;\mathrm{and}\;a_{z}^{*},$$

where $a_{y}^{*}$ and $a_{z}^{*}$ are the typical lengths in the $y^{*}$ and $z^{*}$ directions, respectively.

In order to examine the thermal effects inside the bubble, the energy equation [57] is used (see (11) below) [55], [56], [66], [67]. The pressure and temperature distributions of the gas inside the bubble [68] are not examined directly. Instead, they are treated as the average values of the gas inside bubble. The temperature fluctuation of the gas inside the bubble is considered; however, that of the liquid phase is assumed constant. The bubble oscillation is spherically symmetric. The bubble–bubble interaction [69], [70], [71], [72] is not considered. Bubbles do not coalesce, break up, appear, and disappear. Gas inside the bubbles is composed of only non-condensable gas, and the mass transfer induced by phase change at the bubble–liquid interface [77], [78], [79], which would be important in a scenario of high pressure and long pulses, is not considered. Further, heat transfer at the bubble–liquid interface [80] is not considered. Gas viscosity, viscosity of bulk liquid, and elasticity (from the viewpoint of medical applications) of the liquid phase [72], [81] are not considered. Initially, the bubbly liquid is at rest and spatially uniform, except for the bubble distribution. The initial polydispersity of the bubbles [73], [74], [75], [76] is not considered. Only the bubble distribution is spatially nonuniform in the initial state [53], [54], [55], [56] because medical applications, such as HIFU treatment, often only use the bubbles at the focus.

### 2.2 Basic equations

Although simplified conservation equations based on a mixture model [58] were used in our previous works [55], [56], the volumetric averaged conservation laws of mass and momentum for the gas and liquid phases, based on a two-fluid model [60], are used in this study:

(4) $$\frac{\partial }{\partial t^{*}}(\alpha \rho_{G}^{*})+\nabla^{*}\cdot (\alpha \rho_{G}^{*}u_{G}^{*})=0,$$

(5) $$\frac{\partial }{\partial t^{*}}[(1-\alpha )\rho_{L}^{*}]+\nabla^{*}\cdot [(1-\alpha )\rho_{L}^{*}u_{L}^{*}]=0,$$

(6) $$\frac{\partial }{\partial t^{*}}(\alpha \rho_{G}^{*}u_{G}^{*})+\nabla^{*}\cdot (\alpha \rho_{G}^{*}u_{G}^{*}u_{G}^{*})+\alpha \nabla^{*}p_{G}^{*}=F^{*},$$

(7) $$\frac{\partial }{\partial t^{*}}[(1-\alpha )\rho_{L}^{*}u_{L}^{*}]+\nabla^{*}\cdot [(1-\alpha )\rho_{L}^{*}u_{L}^{*}u_{L}^{*}]+(1-\alpha )\nabla^{*}p_{L}^{*}+P^{*}\nabla^{*}\alpha =-F^{*},$$

where $t^{*}$ is the time, α is the void fraction, $\rho^{*}$ is the density, $u^{*}$ is the velocity vector, $p^{*}$ is the pressure, and $P^{*}$ is the liquid pressure averaged at the bubble–liquid interface [60]. The subscripts G and L denote volume-averaged variables in the gas and liquid phases, respectively. The subscript * denotes a dimensional quantity. The virtual mass force model [60] is introduced as the interfacial momentum transport term $F^{*}$

(8) $$\begin{array}{cc} F^{*}= & -\beta_{1}\alpha \rho_{L}^{*}\left(\frac{D_{G}u_{G}^{*}}{Dt^{*}}-\frac{D_{L}u_{L}^{*}}{Dt^{*}}\right)\\ & -\beta_{2}\rho_{L}^{*}(u_{G}^{*}-u_{L}^{*})\frac{D_{G}\alpha }{Dt^{*}}-\beta_{3}\alpha (u_{G}^{*}-u_{L}^{*})\frac{D_{G}\rho_{L}^{*}}{Dt^{*}}, \end{array}$$

where $\beta_{1}$, $\beta_{2}$, and $\beta_{3}$ are constants and may be set equal to 1/2 for the spherical bubble. For simplicity, the drag force [82], [83], [84], lift force, and gravitation are neglected. The Lagrange derivatives $D_{G}/Dt^{*}$ and $D_{L}/Dt^{*}$ are defined as

(9) $$\frac{D_{G}}{Dt^{*}}=\frac{\partial }{\partial t^{*}}+u_{G}^{*}\cdot \nabla^{*},\;\frac{D_{L}}{Dt^{*}}=\frac{\partial }{\partial t^{*}}+u_{L}^{*}\cdot \nabla^{*}\text{.}$$

The Keller equation for spherical bubble oscillation in a compressible liquid [85] is given as

(10) $$\begin{array}{cc} & \left(1-\frac{1}{c_{L0}^{*}}\frac{D_{G}R^{*}}{Dt^{*}}\right)R^{*}\frac{{D_{G}}^{2}R^{*}}{Dt^{*2}}+\frac{3}{2}\left(1-\frac{1}{3c_{L0}^{*}}\frac{D_{G}R^{*}}{Dt^{*}}\right){\left(\frac{D_{G}R^{*}}{Dt^{*}}\right)}^{2}\\ & =\left(1+\frac{1}{c_{L0}^{*}}\frac{D_{G}R^{*}}{Dt^{*}}\right)\frac{P^{*}}{\rho_{L0}^{*}}+\frac{R^{*}}{\rho_{L0}^{*}c_{L0}^{*}}\frac{D_{G}}{Dt^{*}}(p_{L}^{*}+P^{*}), \end{array}$$

where $R^{*}$ is the bubble radius, $c^{*}$ is the sound speed, and the subscript 0 represents the physical quantity in the initial state.

The energy equation [57] for thermal conduction at the bubble–liquid interface is used to express the thermal effect inside the bubble

(11) $$\frac{D_{G}p_{G}^{*}}{Dt^{*}}=\frac{3}{R^{*}}\left[(\kappa -1)\lambda_{G}^{*}{\left(\frac{\partial T_{G}^{*}}{\partial r_{G}^{*}}\right|}_{r_{G}^{*}=R^{*}}-\kappa p_{G}^{*}\frac{D_{G}R^{*}}{Dt^{*}}\right],$$

where κ is the ratio of specific heats, $\lambda_{G}^{*}$ is the thermal conductivity of the gas phase. $T_{G}^{*}$ is the temperature of the gas phase, and $r_{G}^{*}$ is the radial distance from the center of the bubble. The temperature gradient term $\partial T_{G}^{*}/\partial r_{G}^{*}{|}_{r_{G}^{*}=R^{*}}$, in the first term on the right-hand side of (11), is rewritten using the following four models [66], [67]:

(i) Shimada et al. (SMK) [61]
  (12) $${\left(\frac{\partial T_{G}^{*}}{\partial r_{G}^{*}}\right|}_{r_{G}^{*}=R^{*}}=\frac{5}{4}\frac{T_{0}^{*}-T_{G}^{*}}{R^{*}},$$
(ii) Lertnuwat et al. (LSM) [62]
  (13) $${\left(\frac{\partial T_{G}^{*}}{\partial r_{G}^{*}}\right|}_{r_{G}^{*}=R^{*}}=\frac{T_{0}^{*}-T_{G}^{*}}{\sqrt{2\pi D_{G}^{*}/\omega_{B}^{*}}},$$
(iii) Preston et al. (PCB) [63]
  (14) $${\left(\frac{\partial T_{G}^{*}}{\partial r_{G}^{*}}\right|}_{r_{G}^{*}=R^{*}}=\frac{T_{0}^{*}-T_{G}^{*}}{\left|{\overset{∼}{L}}_{P}^{*}\right|},$$
(iv) Sugiyama et al. (STM) [64]
  (15) ∂TG∗∂rG∗rG∗=R∗=Re(L∼P∗)(T0∗-TG∗)|L∼P∗|2+Im(L∼P∗)ωB∗|L∼P∗|2DGTG∗Dt∗,

where $T_{0}^{*}$ is the initial temperature of the liquid and gas phases, $D_{G}^{*}$ is the thermal diffusivity of the gas phase, and Re and Im denote the real and imaginary parts, respectively. The main features of the temperature models (12), (13), (14), (15) are summarized in our previous study [66]. Note that the STM model (15) incorporates the effect of a phase difference of time between the average temperature inside the bubble and the temperature gradient at the bubble–liquid interface. $\omega_{B}^{*}$ is the linear natural frequency of a single bubble oscillation [64], given as

(16) $$\omega_{B}^{*}=\sqrt{\frac{3\gamma_{e}p_{G0}^{*}-2\sigma^{*}/R_{0}^{*}}{\rho_{L0}^{*}R_{0}^{*2}}-{\left(\frac{2\mu_{e0}^{*}}{\rho_{L0}^{*}R_{0}^{*2}}\right)}^{2}},$$

(17) γe=ReΓN3,

(18) μe0∗=μL∗+ImpG0∗ΓN4ωB∗,

(19) $$\Gamma_{N}=\frac{3\alpha_{N}^{2}\kappa }{\alpha_{N}^{2}+3(\kappa -1)(\alpha_{N}\coth\alpha_{N}-1)},$$

(20) $$\alpha_{N}=\sqrt{\frac{\kappa \omega_{B}^{*}p_{G0}^{*}R_{0}^{*2}}{2(\kappa -1)T_{0}^{*}\lambda_{G}^{*}}}(1+i),$$

where $\gamma_{e}$ is the effective polytropic exponent, $\mu_{e0}^{*}$ is the initial effective viscosity, $\mu_{L}^{*}$ is the liquid viscosity, $\Gamma_{N}$ and $\alpha_{N}$ are complex numbers, and i denotes the imaginary unit. The explicit form of (16) is different from that used in our previous studies [53], [54], [55], [56], [59], [86], [87]. ${\overset{∼}{L}}_{P}^{*}$ in (14), (15) is the complex number with the length dimension, given as

(21) $${\overset{∼}{L}}_{P}^{*}=\frac{R_{0}^{*}(\alpha_{N}^{2}-3\alpha_{N}\coth\alpha_{N}+3)}{\alpha_{N}^{2}(\alpha_{N}\coth\alpha_{N}-1)}\text{.}$$

Note that the form of $\omega_{B}^{*}$ in (16) is valid only for the linear oscillations of symmetric waves with a sinusoidal shape. However, this study investigates the nonlinear propagation, wherein the wave form will develop an asymmetric form. Additionally, the natural frequency of bubble oscillations is affected by the pressure amplitude [88], [89]. Furthermore, bubble–bubble interaction and dual-frequency ultrasound, which are not considered in this study, also affect the natural frequency of bubble oscillations [69], [70], [71], [72], [90], [91], [92], the latter of which can be employed to enhance the effects of bubble oscillations. Since incorporating these effects in the derivation process is quite difficult, the natural linear frequency of bubble oscillations $\omega_{B}^{*}$ is used in this study for simplicity.

To close the set of equations, we use Tait’s equation of state for the liquid phase, the equation of state for the ideal gas, the conservation equation of mass inside the bubble, and the balance of normal stresses across the bubble–liquid interface:

(22) $$p_{L}^{*}=p_{L0}^{*}+\frac{\rho_{L0}^{*}c_{L0}^{*2}}{n}\left[{\left(\frac{\rho_{L}^{*}}{\rho_{L0}^{*}}\right)}^{n}-1\right],$$

(23) $$\frac{p_{G}^{*}}{p_{G0}^{*}}=\frac{\rho_{G}^{*}}{\rho_{G0}^{*}}\frac{T_{G}^{*}}{T_{0}^{*}},$$

(24) $$\frac{\rho_{G}^{*}}{\rho_{G0}^{*}}={\left(\frac{R_{0}^{*}}{R^{*}}\right)}^{3},$$

(25) $$p_{G}^{*}-(p_{L}^{*}+P^{*})=\frac{2\sigma^{*}}{R^{*}}+\frac{4\mu_{L}^{*}}{R^{*}}\frac{D_{G}R^{*}}{Dt^{*}},$$

where *n* is the material constant (e.g., n=7.15 for water). Although (10), (11), (24), (25) were originally derived for a single bubble, this study deals with them as the volumetric averaged equations for liquids containing many bubbles. Hence, the bubble radius $R^{*}(t^{*})$, depending on only the time $t^{*}$ in the original equations, is treated as a volumetric averaged variable and extended as a multivariable function of the temporal and spatial variables, i.e., $R^{*}(x^{*},r^{*},t^{*})$ and $R^{*}(x^{*},y^{*},z^{*},t^{*})$, for the 2D and 3D KZK equations, respectively.

### 2.3 Parameter scaling

As in our previous work [54], [55], [56], [59], [66], [86], [87], the following scaling relations are introduced for the low frequency long wave, based on the nondimensional amplitude ∊(≪1):

(26) $$\frac{U^{*}}{c_{L0}^{*}}\equiv O(\sqrt{∊})\equiv V\sqrt{∊},$$

(27) $$\frac{\omega^{*}}{\omega_{B}^{*}}\equiv O(\sqrt{∊})\equiv \Omega \sqrt{∊},$$

(28) $$\frac{R_{0}^{*}}{L^{*}}\equiv O(\sqrt{∊})\equiv \Delta \sqrt{∊},$$

where $U^{*}$, $\omega^{*}$, and $L^{*}$ are the typical propagation speed, angular frequency, and wavelength, respectively; V, Ω and Δ are the nondimensional constants of O(1).

The assumption of (2), (3) for weakly diffracted waves is rewritten using ∊ (i=r,y,z) [53], [54], [55]

(29) $$\frac{L^{*}}{a_{i}^{*}}\equiv O(\sqrt{∊})\equiv \Gamma_{i}\sqrt{∊},$$

where $\Gamma_{i}$ is the nondimensional constant of O(1) and represents the effect of diffraction for each direction.

The liquid viscosity and initial effective viscosity [66] are nondimensionalized as

(30) $$\frac{\mu_{L}^{*}}{\rho_{L0}^{*}U^{*}L^{*}}=\mu_{L}∊,$$

(31) $$\frac{\mu_{e0}^{*}}{\rho_{L0}^{*}U^{*}L^{*}}=\mu_{e0}∊\text{.}$$

Nondimensionalizations for the energy equations [66] are as follows: for (12)

(32) $$\frac{3(\kappa -1)\lambda_{G}^{*}}{p_{G0}^{*}\omega^{*}R_{0}^{*}}\frac{5}{4}\frac{T_{0}^{*}}{R_{0}^{*}}=\zeta_{\mathrm{SMK}}∊,$$

for (13)

(33) $$\frac{3(\kappa -1)\lambda_{G}^{*}}{p_{G0}^{*}\omega^{*}R_{0}^{*}}\frac{T_{0}^{*}}{\sqrt{2\pi D_{G}^{*}/\omega_{B}^{*}}}=\zeta_{\mathrm{LSM}}∊,$$

for (14)

(34) $$\frac{3(\kappa -1)\lambda_{G}^{*}}{p_{G0}^{*}\omega^{*}R_{0}^{*}}\frac{T_{0}^{*}}{\left|{\overset{∼}{L}}_{P}^{*}\right|}=\zeta_{\mathrm{PCB}}∊,$$

for (15)

(35) 3(κ-1)λG∗pG0∗ω∗R0∗Re(L∼P∗)T0∗|L∼P∗|2=ζSTM1∊,

(36) 3(κ-1)λG∗pG0∗ω∗R0∗ω∗Im(L∼P∗)T0∗ωB∗|L∼P∗|2=ζSTM2∊,

where $\zeta_{\mathrm{SMK}}$, $\zeta_{\mathrm{LSM}}$, $\zeta_{\mathrm{PCB}}$, $\zeta_{\mathrm{STM}1}$ and $\zeta_{\mathrm{STM}2}$ are the constants of O(1); $\zeta_{\mathrm{STM}2}$ in (36) denotes the effect of the phase difference of the time between the average temperature inside the bubble and the temperature gradient at the bubble–liquid interface.

### 2.4 Multiple scale analysis

The independent variables $t^{*}$, $x^{*}$, $r^{*}$, $y^{*}$, and $z^{*}$ are nondimensionalized as

(37) $$t=\frac{t^{*}}{T^{*}},\;x=\frac{x^{*}}{L^{*}},$$

(38) $$\left\{\begin{array}{cc} r=\frac{r^{*}}{L^{*}} & (\mathrm{for}\;2D\;\mathrm{KZK}),\\ y=\frac{y^{*}}{L^{*}},\;z=\frac{z^{*}}{L^{*}} & (\mathrm{for}\;3D\;\mathrm{KZK}), \end{array}\right)$$

where $T^{*}$ is the typical period of a wave and is related to the wave speed $U^{*}$ and wave length $L^{*}$ by $U^{*}=L^{*}/T^{*}$. Next, the nondimensionalized time *t* and the spatial coordinate *x*, for the direction of the ultrasound propagation, are expanded to the near field and far field using the nondimensional wave amplitude ∊, for both 2D and 3D KZK equations [65]

(39) $$t_{0}=t,\;x_{0}=x\;(\mathrm{near}\;\mathrm{field}),$$

(40) $$t_{1}=∊t,\;x_{1}=∊x\;(\mathrm{far}\;\mathrm{field})\text{.}$$

Owing to the assumption of weakly diffracted waves in (2), the spatial variations in the radial distance from the *x* axis appear only in the far field and not the near field [53], [54], [55], [56], [87]. Then, the spatial coordinates *r*, *y*, or *z* for the radial distance from *x* are defined only for the far field as

(41) $$\left\{\begin{array}{cc} r_{1/2}=\sqrt{∊}\Gamma_{r}r & (\mathrm{for}\;2D\;\mathrm{KZK}),\\ y_{1/2}=\sqrt{∊}\Gamma_{y}y,\;z_{1/2}=\sqrt{∊}\Gamma_{z}z & (\mathrm{for}\;3D\;\mathrm{KZK})\text{.} \end{array}\right)$$

Although the relationship in (41) for 2D KZK is the same as in our previous study [53], [54], [55], [56], [87], the relationship for 3D KZK is introduced in this study. For (41), the relationships are taken as

(42) $$\left\{\begin{array}{c} r=\frac{a_{r}^{*}}{L^{*}}\frac{r^{*}}{a_{r}^{*}}=\frac{r_{1/2}}{\sqrt{∊}\Gamma_{r}}\left(r_{1/2}\equiv \frac{r^{*}}{a_{r}^{*}},\;\mathrm{for}\;2D\;\mathrm{KZK}\right),\\ y=\frac{a_{y}^{*}}{L^{*}}\frac{y^{*}}{a_{y}^{*}}=\frac{y_{1/2}}{\sqrt{∊}\Gamma_{y}},\;z=\frac{a_{z}^{*}}{L^{*}}\frac{z^{*}}{a_{z}^{*}}=\frac{z_{1/2}}{\sqrt{∊}\Gamma_{z}}\\ \left(y_{1/2}\equiv \frac{y^{*}}{a_{y}^{*}},\;z_{1/2}\equiv \frac{z^{*}}{a_{z}^{*}},\;\mathrm{for}\;3D\;\mathrm{KZK}\right)\text{.} \end{array}\right)$$

All the dependent variables are regarded as functions of the extended independent variables of (37), (38), (39), (40), (41), (42). The differential operators are thus expanded as [65]

(43) $$\frac{\partial }{\partial t}=\frac{\partial }{\partial t_{0}}+∊\frac{\partial }{\partial t_{1}},$$

(44) $$\frac{\partial }{\partial x}=\frac{\partial }{\partial x_{0}}+∊\frac{\partial }{\partial x_{1}},$$

(45) $$\left\{\begin{array}{cc} \frac{\partial }{\partial r}=\sqrt{∊}\Gamma_{r}\frac{\partial }{\partial r_{1/2}} & (\mathrm{for}\;2D\;\mathrm{KZK}),\\ \frac{\partial }{\partial y}=\sqrt{∊}\Gamma_{y}\frac{\partial }{\partial y_{1/2}},\;\frac{\partial }{\partial z}=\sqrt{∊}\Gamma_{z}\frac{\partial }{\partial z_{1/2}} & (\mathrm{for}\;3D\;\mathrm{KZK})\text{.} \end{array}\right)$$

The dependent variables are expanded in powers of ∊

(46) $$\frac{T_{G}^{*}}{T_{0}^{*}}=1+∊T_{G1}+{∊}^{2}T_{G2}+O({∊}^{3}),$$

(47) $$\frac{R^{*}}{R_{0}^{*}}=1+∊R_{1}+{∊}^{2}R_{2}+O({∊}^{3}),$$

(48) $$\frac{p_{L}^{*}}{\rho_{L0}^{*}U^{*2}}=p_{L0}+∊p_{L1}+{∊}^{2}p_{L2}+O({∊}^{3}),$$

(49) $$\frac{u_{G}^{*}}{U^{*}}=∊u_{G1}+{∊}^{2}u_{G2}+O({∊}^{3}),$$

(50) $$\frac{u_{L}^{*}}{U^{*}}=∊u_{L1}+{∊}^{2}u_{L2}+O({∊}^{3}),$$

where $u^{*}$ is the velocity component in the $x^{*}$ direction. The velocity components in the $r^{*}$, $y^{*}$, and $z^{*}$ directions are $v_{r}^{*}$, $v_{y}^{*}$, and $v_{z}^{*}$, respectively. The velocity components are expanded for the gas and liquid phases as

(51) $$\left\{\begin{array}{cc} \frac{v_{rG}^{*}}{U^{*}}={∊}^{3/2}v_{rG1}+{∊}^{5/2}v_{rG2}+O({∊}^{7/2}) & (\mathrm{for}\;2D\;\mathrm{KZK}),\\ \frac{v_{yG}^{*}}{U^{*}}={∊}^{3/2}v_{yG1}+{∊}^{5/2}v_{yG2}+O({∊}^{7/2}),\\ \frac{v_{zG}^{*}}{U^{*}}={∊}^{3/2}v_{zG1}+{∊}^{5/2}v_{zG2}+O({∊}^{7/2}) & (\mathrm{for}\;3D\;\mathrm{KZK}), \end{array}\right)$$

(52) $$\left\{\begin{array}{cc} \frac{v_{rL}^{*}}{U^{*}}={∊}^{3/2}v_{rL1}+{∊}^{5/2}v_{rL2}+O({∊}^{7/2}) & (\mathrm{for}\;2D\;\mathrm{KZK}),\\ \frac{v_{yL}^{*}}{U^{*}}={∊}^{3/2}v_{yL1}+{∊}^{5/2}v_{yL2}+O({∊}^{7/2}),\\ \frac{v_{zL}^{*}}{U^{*}}={∊}^{3/2}v_{zL1}+{∊}^{5/2}v_{zL2}+O({∊}^{7/2}) & (\mathrm{for}\;3D\;\mathrm{KZK})\text{.} \end{array}\right)$$

The magnitude of the variations in the velocity components in the $r^{*}$, $y^{*}$, and $z^{*}$ directions vertical to the $x^{*}$ axis are assumed to be smaller than those in the $x^{*}$ direction [53], [54], [55], [56], [87]. Then, the expansions of $v_{r}^{*}$, $v_{y}^{*}$, and $v_{z}^{*}$ begin with a higher order than that of $u^{*}$ in (49), (50), (51), (52).

The density of the gas phase at the initial state is assumed to be significantly smaller than that of the liquid phase

(53) $$\frac{\rho_{G0}^{*}}{\rho_{L0}^{*}}\equiv O({∊}^{3})\text{.}$$

The nondimensional pressures of the liquid and gas phases at the initial state are defined as

(54) $$p_{L0}=\frac{p_{L0}^{*}}{\rho_{L0}^{*}U^{*2}}\equiv O(1),\;p_{G0}=\frac{p_{G0}^{*}}{\rho_{L0}^{*}U^{*2}}\equiv O(1)\text{.}$$

The expansion of the liquid density is given as

(55) $$\frac{\rho_{L}^{*}}{\rho_{L0}^{*}}=1+{∊}^{2}\rho_{L1}+{∊}^{3}\rho_{L2}+O({∊}^{4}),$$

which is determined using (22), (26), and (48) [86].

To consider the effect of the weak nonuniformity of the bubble number density at the initial state, the void fraction α is expanded as [53], [54], [55], [56], [87]

(56) $$\frac{\alpha }{\alpha_{0}}=1+∊[\alpha_{1}+\delta (x_{1})]+{∊}^{2}\alpha_{2}+O({∊}^{3}),$$

where δ is the known variable and denotes the nonuniformity of the bubble number density at the initial state. The effect of the nonuniformity of the bubble number density is assumed to appear only at the far field from the sound source, thus δ is only a function of $x_{1}$ [53], [54], [55], [56], [87].

## 3 Results of theoretical analysis

The scaling relations of (26), (27), (28), (29), (30), (31), (32), (33), (34), (35), (36), the differential operators of (43), (44), (45), and the expansions of the dependent variables in (46), (47), (48), (49), (50), (51), (52), (53), (54), (55), (56) are substituted into the basic equations (4), (5), (6), (7), (8), (9), (10), (11), (12), (13), (14), (15), (16), (17), (18), (19), (20), (21), (22), (23), (24), (25). At each order of ∊, approximated equations are obtained.

### 3.1 First order of approximation

From the approximations of O(∊), linear equations are obtained for (4), (5), (6), (7), (10), and (11) as

(57) $$\frac{\partial \alpha_{1}}{\partial t_{0}}-3\frac{\partial R_{1}}{\partial t_{0}}+\frac{\partial u_{G1}}{\partial x_{0}}=0,$$

(58) $$\alpha_{0}\frac{\partial \alpha_{1}}{\partial t_{0}}-(1-\alpha_{0})\frac{\partial u_{L1}}{\partial x_{0}}=0,$$

(59) $$\beta_{1}\frac{\partial u_{G1}}{\partial t_{0}}-\beta_{1}\frac{\partial u_{L1}}{\partial t_{0}}-3p_{G0}\frac{\partial R_{1}}{\partial x_{0}}+p_{G0}\frac{\partial T_{G1}}{\partial x_{0}}=0,$$

(60) $$(1-\alpha_{0}+\beta_{1}\alpha_{0})\frac{\partial u_{L1}}{\partial t_{0}}-\beta_{1}\alpha_{0}\frac{\partial u_{G1}}{\partial t_{0}}+(1-\alpha_{0})\frac{\partial p_{L1}}{\partial x_{0}}=0,$$

(61) $$-\frac{\Delta^{2}}{\Omega^{2}}R_{1}-p_{L1}+p_{G0}T_{G1}+3(\gamma_{e}-1)p_{G0}R_{1}+\frac{4\mu_{e0}^{2}}{\Delta^{2}}=0,$$

(62) $$\frac{\partial T_{G1}}{\partial t_{0}}+3(\kappa -1)\frac{\partial R_{1}}{\partial t_{0}}=0\text{.}$$

These equations are different from our previous study [55], [56], which was based on the mixture model equations. (57), (58), (59), (60), (61), (62) are combined into a single equation for $R_{1}$ as

(63) $$\frac{\partial^{2}R_{1}}{\partial t_{0}^{2}}-v_{P}^{2}\frac{\partial^{2}R_{1}}{\partial x_{0}^{2}}=0,$$

where the phase velocity $v_{P}$ is given as

(64) $$v_{P}=\sqrt{\frac{3\alpha_{0}(1-\alpha_{0}+\beta_{1})\kappa p_{G0}-(1-\alpha_{0})\beta_{1}s_{4}}{3(1-\alpha_{0})\beta_{1}\alpha_{0}}},$$

and $s_{4}$ is given as

(65) $$s_{4}=3(\gamma_{e}-\kappa )p_{G0}-\frac{\Delta^{2}}{\Omega^{2}}\text{.}$$

For simplicity, we set $v_{P}\equiv 1$, and the typical wave speed $U^{*}$ is given as

(66) $$U^{*}=\sqrt{\frac{3\alpha_{0}(1-\alpha_{0}+\beta_{1})\kappa p_{G0}^{*}/\rho_{L0}^{*}-(1-\alpha_{0})\beta_{1}s_{4}^{*}}{3(1-\alpha_{0})\beta_{1}\alpha_{0}}},$$

where $s_{4}^{*}$ is given as

(67) $$s_{4}^{*}=3(\gamma_{e}-\kappa )\frac{p_{G0}^{*}}{\rho_{L0}^{*}}-R_{0}^{*2}\omega_{B}^{*2}\text{.}$$

Next, the following variable transformation is introduced:

(68) $$\phi_{0}=t_{0}-x_{0}\text{.}$$

Then, we obtain the equation describing the propagation of right-running waves as

(69) $$\frac{\partial R_{1}}{\partial t_{0}}+\frac{\partial R_{1}}{\partial x_{0}}=0\text{.}$$

By introducing the variable transform (68) into the approximated (57), (58), (59), (60), (61), (62), all dependent variables can be written in terms of $R_{1}=f$ as

(70) $$\begin{array}{cc} & \alpha_{1}=s_{1}f,\;u_{G1}=s_{2}f,\;u_{L1}=s_{3}f,\\ & p_{L1}=s_{4}f,\;T_{G1}=s_{5}f, \end{array}$$

with

(71) $$s_{1}=3(1-\alpha_{0})-\frac{\kappa {\left(1-\alpha_{0}\right)}^{2}(3\alpha_{0}-\Delta^{2}/\Omega^{2})}{\alpha_{0}(1-\alpha_{0}+\beta_{1})\kappa -(\gamma_{e}-\kappa )(1-\alpha_{0})\beta_{1}},$$

(72) $$s_{2}=s_{1}-3,$$

(73) $$s_{3}=-\frac{\alpha_{0}s_{1}}{1-\alpha_{0}},$$

(74) $$s_{5}=-3(\kappa -1)\text{.}$$

### 3.2 Approximation of radial direction

At the approximations of $O({∊}^{2/3})$, the equations obtained from the radial directions of the momentum conservation equations (6), (7) are

(75) $$\left\{\begin{array}{cc} \beta_{1}\frac{\partial v_{rG1}}{\partial t_{0}}-\beta_{1}\frac{\partial v_{rL1}}{\partial t_{0}}-3p_{G0}\Gamma_{r}\frac{\partial R_{1}}{\partial r_{1/2}}+p_{G0}\Gamma_{r}\frac{\partial T_{G1}}{\partial r_{1/2}}=0\\ & (\mathrm{for}\;2D\;\mathrm{KZK}),\\ \beta_{1}\frac{\partial v_{yG1}}{\partial t_{0}}-\beta_{1}\frac{\partial v_{yL1}}{\partial t_{0}}-3p_{G0}\Gamma_{y}\frac{\partial R_{1}}{\partial y_{1/2}}+p_{G0}\Gamma_{y}\frac{\partial T_{G1}}{\partial y_{1/2}}=0,\\ \beta_{1}\frac{\partial v_{zG1}}{\partial t_{0}}-\beta_{1}\frac{\partial v_{zL1}}{\partial t_{0}}-3p_{G0}\Gamma_{z}\frac{\partial R_{1}}{\partial z_{1/2}}+p_{G0}\Gamma_{z}\frac{\partial T_{G1}}{\partial z_{1/2}}=0\\ & (\mathrm{for}\;3D\;\mathrm{KZK}), \end{array}\right)$$

(76) $$\left\{\begin{array}{cc} (1-\alpha_{0}+\beta_{1}\alpha_{0})\frac{\partial v_{rL1}}{\partial t_{0}}-\beta_{1}\alpha_{0}\frac{\partial v_{rG1}}{\partial t_{0}}+\Gamma_{r}(1-\alpha_{0})\frac{\partial p_{L1}}{\partial r_{1/2}}=0\\ & (\mathrm{for}\;2D\;\mathrm{KZK}),\\ (1-\alpha_{0}+\beta_{1}\alpha_{0})\frac{\partial v_{yL1}}{\partial t_{0}}-\beta_{1}\alpha_{0}\frac{\partial v_{yG1}}{\partial t_{0}}+\Gamma_{y}(1-\alpha_{0})\frac{\partial p_{L1}}{\partial y_{1/2}}=0,\\ (1-\alpha_{0}+\beta_{1}\alpha_{0})\frac{\partial v_{zL1}}{\partial t_{0}}-\beta_{1}\alpha_{0}\frac{\partial v_{zG1}}{\partial t_{0}}+\Gamma_{z}(1-\alpha_{0})\frac{\partial p_{L1}}{\partial z_{1/2}}=0\\ & (\mathrm{for}\;3D\;\mathrm{KZK})\text{.} \end{array}\right)$$

The independent variables $r_{1/2}$, $y_{1/2}$, and $z_{1/2}$ first appear here. The variable transformation (68) is introduced into (75), (76), and then we obtain the relations (i  = G, L) of

(77) $$\left\{\begin{array}{cc} \frac{\partial v_{\mathrm{ri}1}}{\partial \phi_{0}}=\Gamma_{r}C_{i}\frac{\partial f}{\partial r_{1/2}} & (\mathrm{for}\;2D\;\mathrm{KZK}),\\ \frac{\partial v_{\mathrm{yi}1}}{\partial \phi_{0}}=\Gamma_{y}C_{i}\frac{\partial f}{\partial y_{1/2}},\;\frac{\partial v_{\mathrm{zi}1}}{\partial \phi_{0}}=\Gamma_{z}C_{i}\frac{\partial f}{\partial z_{1/2}}\; & (\mathrm{for}\;3D\;\mathrm{KZK}), \end{array}\right)$$

where the constant coefficients $C_{i}$ (i=G,L) are given as

(78) $$C_{G}=\frac{\kappa (\alpha_{0}\beta_{1}+1-\alpha_{0})(3\alpha_{0}-\Delta^{2}/\Omega^{2})}{\alpha_{0}(1-\alpha_{0}+\beta_{1})\kappa -(\gamma_{e}-\kappa )(1-\alpha_{0})\beta_{1}}-s_{4},$$

(79) $$C_{L}=\frac{\kappa \alpha_{0}\beta_{1}(3\alpha_{0}-\Delta^{2}/\Omega^{2})}{\alpha_{0}(1-\alpha_{0}+\beta_{1})\kappa -(\gamma_{e}-\kappa )(1-\alpha_{0})\beta_{1}}-s_{4}\text{.}$$

### 3.3 Second order of approximation

From the approximations of $O({∊}^{2})$, equations are obtained for (4), (5), (6), (7), 10, and 11 as

(80) $$\frac{\partial \alpha_{2}}{\partial t_{0}}-3\frac{\partial R_{2}}{\partial t_{0}}+\frac{\partial u_{G2}}{\partial x_{0}}=K_{1},$$

(81) $$\alpha_{0}\frac{\partial \alpha_{2}}{\partial t_{0}}-(1-\alpha_{0})\frac{\partial u_{L2}}{\partial x_{0}}=K_{2},$$

(82) $$\beta_{1}\frac{\partial u_{G2}}{\partial t_{0}}-\beta_{1}\frac{\partial u_{L2}}{\partial t_{0}}-3p_{G0}\frac{\partial R_{2}}{\partial x_{0}}+p_{G0}\frac{\partial T_{G2}}{\partial x_{0}}=K_{3},$$

(83) $$(1-\alpha_{0}+\beta_{1}\alpha_{0})\frac{\partial u_{L2}}{\partial t_{0}}-\beta_{1}\alpha_{0}\frac{\partial u_{G2}}{\partial t_{0}}+(1-\alpha_{0})\frac{\partial p_{L2}}{\partial x_{0}}=K_{4},$$

(84) $$-\frac{\Delta^{2}}{\Omega^{2}}R_{2}-p_{L2}+p_{G0}T_{G2}+3(\gamma_{e}-1)p_{G0}R_{2}=K_{5},$$

(85) $$\frac{\partial T_{G2}}{\partial t_{0}}+3(\kappa -1)\frac{\partial R_{2}}{\partial t_{0}}=K_{6},$$

where the explicit forms of the inhomogeneous terms $K_{i}\;(i=1,2,3,4,5,6)$ are presented in Appendix A. Equations (80), (81), (82), (83), (84), (85) are combined into the single equation

(86) $$\frac{\partial^{2}R_{2}}{\partial t_{0}^{2}}-\frac{\partial^{2}R_{2}}{\partial x_{0}^{2}}=K\text{.}$$

The inhomogeneous term *K* is given as

(87) $$\begin{array}{cc} K & =-\frac{1}{3}\frac{\partial K_{1}}{\partial t_{0}}+\frac{1}{3\alpha_{0}}\frac{\partial K_{2}}{\partial t_{0}}+\frac{1-\alpha_{0}+\beta_{1}}{3\beta_{1}(1-\alpha_{0})}\frac{\partial K_{3}}{\partial x_{0}}\\ & +\frac{1}{3\alpha_{0}(1-\alpha_{0})}\frac{\partial K_{4}}{\partial x_{0}}+\frac{1}{3\alpha_{0}}\frac{\partial^{2}K_{5}}{\partial x_{0}^{2}}\\ & -\frac{\alpha_{0}(1-\alpha_{0})+\beta_{1}}{3\beta_{1}\alpha_{0}(1-\alpha_{0})}p_{G0}\int \frac{\partial^{2}K_{6}}{\partial x_{0}^{2}}dt_{0}\text{.} \end{array}$$

K=0 is imposed as the solvable condition for (86) [53], [54], [86], [87], obtaining the relation

(88) $$\begin{array}{cc} 2\frac{\partial }{\partial \phi_{0}} & \left\{\frac{\partial f}{\partial t_{1}}+\frac{\partial f}{\partial x_{1}}+[\Pi_{0}+\Pi_{4}\delta (x_{1})]\frac{\partial f}{\partial \phi_{0}}\right)\\ & \left(+\Pi_{1}f\frac{\partial f}{\partial \phi_{0}}+\Pi_{21}\frac{\partial^{2}f}{\partial \phi_{0}^{2}}+\Pi_{22}f+\Pi_{3}\frac{\partial^{3}f}{\partial \phi_{0}^{3}}\right\}\\ & =\left\{\begin{array}{cc} \Gamma_{r}^{2}\frac{1}{r_{1/2}}\frac{\partial }{\partial r_{1/2}}\left(r_{1/2}\frac{\partial f}{\partial r_{1/2}}\right) & (\mathrm{for}\;2D\;\mathrm{KZK}),\\ \Gamma_{y}^{2}\frac{\partial^{2}f}{\partial y_{1/2}^{2}}+\Gamma_{z}^{2}\frac{\partial^{2}f}{\partial z_{1/2}^{2}} & (\mathrm{for}\;3D\;\mathrm{KZK})\text{.} \end{array}\right) \end{array}$$

The differential operators (43), (44), (45), the relation in the near field (69), the relation in the radial direction (77), (78), (79) and (88) are combined as

(89) $$\begin{array}{cc} & \frac{\partial }{\partial t}\left[\frac{\partial f}{\partial t}+\frac{\partial f}{\partial x}+∊\left\{[\Pi_{0}+\Pi_{4}\delta (x_{1})]\frac{\partial f}{\partial t}\right)\right)\\ & \;\;\left(\left(+\Pi_{1}f\frac{\partial f}{\partial t}+\Pi_{21}\frac{\partial^{2}f}{\partial t^{2}}+\Pi_{22}f+\Pi_{3}\frac{\partial^{3}f}{\partial t^{3}}\right\}\right]\\ & =\left\{\begin{array}{cc} \frac{1}{2r}\frac{\partial }{\partial r}\left(r\frac{\partial f}{\partial r}\right) & (\mathrm{for}\;2D\;\mathrm{KZK}),\\ \frac{1}{2}\left(\frac{\partial^{2}f}{\partial y^{2}}+\frac{\partial^{2}f}{\partial z^{2}}\right) & (\mathrm{for}\;3D\;\mathrm{KZK})\text{.} \end{array}\right) \end{array}$$

Finally, the 2D and 3D KZK equations are obtained:

(90) $$\frac{\partial }{\partial \tau }\left(\frac{\partial f}{\partial X}+\Pi_{1}f\frac{\partial f}{\partial \tau }+\Pi_{21}\frac{\partial^{2}f}{\partial \tau^{2}}+\Pi_{22}f+\Pi_{3}\frac{\partial^{3}f}{\partial \tau^{3}}\right)=\Delta_{⊥}f,$$

where $\Delta_{⊥}$ is the Laplacian with respect to the radial direction:

(91) $$\Delta_{⊥}f=\left\{\begin{array}{cc} \frac{\Gamma_{r}^{2}}{2}\left(\frac{\partial^{2}f}{\partial R^{2}}+\frac{1}{R}\frac{\partial f}{\partial R}\right) & (\mathrm{for}\;2D\;\mathrm{KZK}),\\ \frac{1}{2}\left(\Gamma_{y}^{2}\frac{\partial^{2}f}{\partial Y^{2}}+\Gamma_{z}^{2}\frac{\partial^{2}f}{\partial Z^{2}}\right) & (\mathrm{for}\;3D\;\mathrm{KZK})\text{.} \end{array}\right)$$

The following variable transformations are used:

(92) $$\tau =t-\{1+∊[\Pi_{0}+\Pi_{4}\delta (x_{1})]\}x,$$

(93) X=∊x,

(94) $$\left\{\begin{array}{cc} R=\sqrt{∊}\Gamma_{r}r & (\mathrm{for}\;2D\;\mathrm{KZK}),\\ Y=\sqrt{∊}\Gamma_{y}y,\;Z=\sqrt{∊}\Gamma_{z}z & (\mathrm{for}\;3D\;\mathrm{KZK}), \end{array}\right)$$

where τ is the retarded time.

### 3.4 Coefficients of the KZK equation

**Fig. 2:**
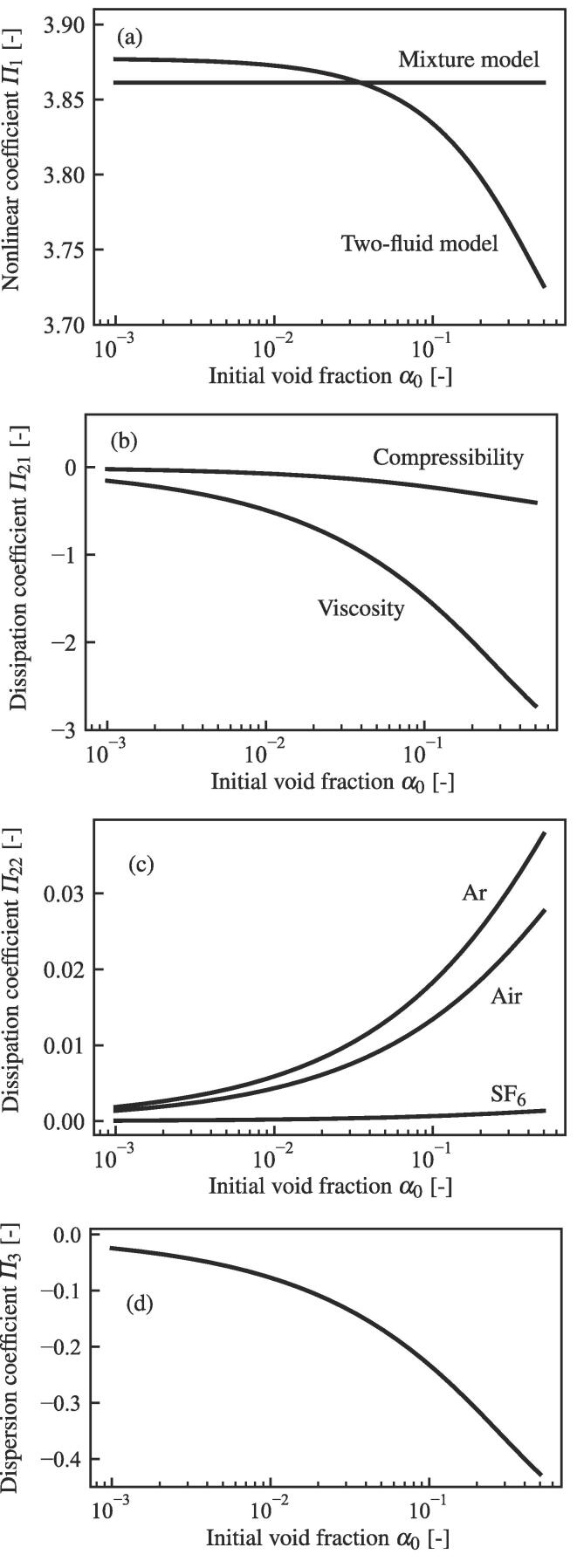
(a) Nonlinear, (b) and (c) dissipation and (d) dispersion coefficients versus the initial void fraction $\alpha_{0}$ for the case of $R_{0}^{*}=1.0$ μm, $f^{*}=100$ kHz, $p_{L0}^{*}=101325\;\mathrm{Pa}$, $\rho_{L0}^{*}=1000\;\mathrm{kg}/m^{3}$, $c_{L0}^{*}=1500\;m/s$, $\sigma^{*}=0.0728\;N/m$ and $\mu_{L}^{*}=0.001\;\mathrm{Pa}\cdot s$. The gas inside the bubble is air (κ=1.40, $\lambda_{G}^{*}=0.025\;W/(m\cdot K)$) in (a), (b), and (d). In (c), the cases of Ar (κ=1.67, $\lambda_{G}^{*}=0.016\;W/(m\cdot K)$) and ${\mathrm{SF}}_{6}$ (κ=1.09, $\lambda_{G}^{*}=0.013\;W/(m\cdot K)$) are also shown.

In the KZK equation (90), $\Pi_{0}$ and $\Pi_{4}$ are the advection coefficients, $\Pi_{1}$ is the nonlinear coefficient, $\Pi_{21}$ and $\Pi_{22}$ are the dissipation coefficients, and $\Pi_{3}$ is the dispersion coefficient. The right hand side of the KZK equation (90) represents the diffraction (focusing) effect, and these terms are expressed differently for the 2D and 3D KZK equations. The nonuniformity of the bubble number density δ appears only in the variable transformation (92) with the advection coefficient $\Pi_{4}$, thus, it only influences the advection effect [53], [54], [55], [56], [87]. Figure 2 shows the dependence of the (a) nonlinear, (b) and (c) dissipation, and (d) dispersion coefficients versus the initial void fraction $\alpha_{0}$ for the normal condition of the air–water system. Note that the effect of bubble–bubble interaction [69], [70], [71], [72] would be dominant when the void fraction increases; however, this effect is not considered in this study.

The nonlinear coefficient $\Pi_{1}$ is given as

(95) $$\begin{array}{cc} \Pi_{1}=\frac{1}{6} & \left\{k_{1}-\frac{k_{2}}{\alpha_{0}}+\frac{1-\alpha_{0}+\beta_{1}}{\beta_{1}(1-\alpha_{0})}k_{3}+\frac{k_{4}}{\alpha_{0}(1-\alpha_{0})}\right)\\ & \left(-\frac{2\Delta^{2}}{\alpha_{0}\Omega^{2}}k_{5}+\frac{[\alpha_{0}(1-\alpha_{0})+\beta_{1}]p_{G0}}{\beta_{1}\alpha_{0}(1-\alpha_{0})}k_{6}\right\}, \end{array}$$

with

(96) $$k_{1}=-6(2-s_{1})-2s_{2}(3-s_{1}),$$

(97) $$k_{2}=2\alpha_{0}s_{1}s_{3},$$

(98) k^=(β1+β2)(s2-s3)s1-β1(s22-s32),

(99) k3=-k^-[3(s1-4)+s5(6-s1)]pG0,

(100) k4=α0k^-α0s1s4+2(1-α0)s32+2α0s1s3,

(101) $$k_{5}=-1-\frac{3(2-\gamma_{e}-s_{5})p_{G0}\Omega^{2}}{\Delta^{2}},$$

(102) $$k_{6}=-3(\kappa -1)(3\kappa -4+2s_{5})\text{.}$$

In Fig. 2 (a), the nonlinear coefficient $\Pi_{1}$ in our previous study [55], [56] derived from the mixture model, does not depend on $\alpha_{0}$. However, it does depend on $\alpha_{0}$ in this study because the two-fluid model [60] is used.

Furthermore, $\Pi_{21}$, $\Pi_{3}$, and $\Pi_{4}$ are given as

(103) $$\Pi_{21}=-\frac{1}{6\alpha_{0}}\left[4\mu_{L}+3(\kappa -\gamma_{e})V\Delta p_{G0}+\frac{V\Delta^{3}}{\Omega^{2}}\right],$$

(104) $$\Pi_{3}=-\frac{\Delta^{2}}{6\alpha_{0}},$$

(105) $$\Pi_{4}=-\frac{\alpha_{0}s_{1}}{6{\left(1-\alpha_{0}\right)}^{2}}-\frac{s_{4}}{6(1-\alpha_{0})\alpha_{0}}\text{.}$$

The first term of the dissipation coefficient $\Pi_{21}$ (103) relates to the interfacial viscosity, the second and third terms relate to the liquid compressibility. Fig. 2 (b) shows the dependence of $\Pi_{21}$ divided into the effects of the interfacial viscosity and the liquid compressibility, showing that the interfacial viscosity is dominant. The viscous effects of bulk liquid were considered in our previous study [55], [56]; however, these effects are omitted in this study because the basic equations based on the two-fluid model that incorporate the effect of the viscosity of the bulk liquid have yet to be developed.

The advection coefficient $\Pi_{0}$ depends on which temperature gradient model from (12), (13), (14), (15) is used

(i) For (12), (14),
  (106) $$\Pi_{0}=-\frac{(1-\alpha_{0})V^{2}s_{4}}{6\alpha_{0}}+\frac{s_{1}}{3(1-\alpha_{0})}\frac{2\mu_{e0}^{2}}{\Delta^{2}}-\frac{1}{3\alpha_{0}}\frac{2\mu_{e0}^{2}}{\Delta^{2}},$$
(ii) For (15),
  (107) $$\begin{array}{cc} \Pi_{0}= & -\frac{(1-\alpha_{0})V^{2}s_{4}}{6\alpha_{0}}+\frac{s_{1}}{3(1-\alpha_{0})}\frac{2\mu_{e0}^{2}}{\Delta^{2}}-\frac{1}{3\alpha_{0}}\frac{2\mu_{e0}^{2}}{\Delta^{2}}\\ & -\frac{3(\kappa -1)}{2}\frac{[\alpha_{0}(1-\alpha_{0})+\beta_{1}]p_{G0}}{3\beta_{1}\alpha_{0}(1-\alpha_{0})}\zeta_{\mathrm{STM}2}\text{.} \end{array}$$

From (107), a phase difference between the average temperature inside the bubble and the temperature gradient at the bubble–liquid interface affects the advection effect. The dissipation coefficient $\Pi_{22}$ is given for each temperature gradient model in (12), (13), (14), (15) (j  =  SMK, LSM, PCB, STM1) as

(108) $$\Pi_{22}=\frac{\kappa -1}{2}\frac{[\alpha_{0}(1-\alpha_{0})+\beta_{1}]p_{G0}}{\beta_{1}\alpha_{0}(1-\alpha_{0})}\zeta_{j}\text{.}$$

Fig. 2 (c) shows the dependence of the dissipation coefficient $\Pi_{22}$ for cases where the gas inside the bubble is air, Argon (Ar) and sulfur hexafluoride (${\mathrm{SF}}_{6}$). Shimada et al. [61] (SMK) temperature gradient model in (12) is used. Although $\Pi_{21}$ is the dissipation coefficient owing to the interfacial viscosity and the liquid compressibility, $\Pi_{22}$ is the dissipation coefficient without differentiation owing to the thermal conduction of the gas inside the bubble. Then, Fig. 2 shows that the effects of thermal conduction decrease from Argon to air to ${\mathrm{SF}}_{6}$. This result agrees with the analysis for the single-bubble oscillation proposed by Matsumoto et al. [93].

### 3.5 Comparison with original KZK equation

The original KZK equation [6], [7] for pure liquid in the dimensional form is given as follows:

(109) $$\frac{\partial }{\partial \tau^{*}}\left(\frac{\partial p^{*}}{\partial X^{*}}-\frac{\beta }{c_{L0}^{*3}\rho_{0}^{*}}p^{*}\frac{\partial p^{*}}{\partial \tau^{*}}-\frac{\delta^{*}}{2c_{L0}^{*3}}\frac{\partial^{2}p^{*}}{\partial \tau^{*2}}\right)=\frac{c_{L0}^{*}}{2}\Delta_{⊥}^{*}p^{*},$$

where β and $\delta^{*}$ are the nonlinear and dissipation coefficients, respectively. The most important difference between the present KZK equation (90) and the original (109) is that the former has the dispersion term as a third-order partial derivative. Further, in the present equation (90), the dissipation term without differentiation was discovered due to the bubble oscillations and the thermal conduction of gas inside bubbles.

### 3.6 Limitation of present model

The present KZK equation (90) is derived by perturbation analysis up to second-order approximation, in which nonlinear propagation is included as the term with the coefficient $\Pi_{1}$. However, the viscosity, compressibility, and thermal conductivity dissipation terms are represented only in linear form. Although the nonlinear dissipation effects [94], [95], [96], [97] will be important particularly when the pressure amplitude increases, they have been omitted in the present equation. Perturbation analysis with greater than third-order approximation will be necessary to incorporate the nonlinear dissipation effects and will be performed in a forthcoming study.

## 4 Numerical example

### 4.1 Method

We numerically solve the newly obtained 2D KZK equation (90) using the FDTD method developed by Lee and Hamilton [22], [23]; this scheme has been widely utilized for the problem of focused ultrasound radiated by a circular sound source in pure water [9], [13], [14], [15], [24], [26], [27], [28], [29], [30], [31]. For consistency between theoretical studies [53], [54], [55], [56], [87] and the numerical studies of Lee and Hamilton [22], [23], a scaling relation is assumed as

(110) $$\frac{L^{*}}{d_{f}^{*}}\equiv O(∊)\equiv \chi ∊,$$

where $d_{f}^{*}$ is the focal length, and χ is the nondimensional constant of O(1). Hereafter, we set χ=1 for simplicity. For numerical calculations, the KZK equation (90) is rewritten by the definite integral regarding the retarded time τ, and the dependent variable is changed from the nondimensionalized bubble radius fluctuation *f* to the nondimensionalized temperature fluctuation of the gas inside the bubble $T_{G1}$ through (70) as

(111) $$\begin{array}{cc} \frac{\partial T_{G1}}{\partial X}= & \frac{1}{4G_{r}}\int_{-\infty }^{\tau }\left(\frac{\partial^{2}T_{G1}}{\partial R^{2}}+\frac{1}{R}\frac{\partial T_{G1}}{\partial R}\right)d\tau^{\prime }-\frac{\Pi_{1}}{s_{5}}T_{G1}\frac{\partial T_{G1}}{\partial \tau }\\ & -\Pi_{21}\frac{\partial^{2}T_{G1}}{\partial \tau^{2}}-\Pi_{22}T_{G1}-\Pi_{3}\frac{\partial^{3}T_{G1}}{\partial \tau^{3}}\text{.} \end{array}$$

In previous studies of focused ultrasound in a pure liquid, the KZK equation is derived in the form describing the distribution of liquid pressure. However, in this study, we succeeded in describing the temperature distribution of the gas inside the bubble $T_{G1}$ by introducing the energy equation (11). In (111), $G_{r}$ is the focusing gain, which is given as

(112) $$G_{r}=\frac{\omega^{*}a_{r}^{*2}}{2U^{*}d_{f}^{*}}\;\left(=\frac{1}{2}\frac{a_{r}^{*2}}{L^{*2}}\frac{L^{*}}{d_{f}^{*}}=\frac{1}{2}\frac{1}{\Gamma_{r}^{2}∊}∊=\frac{1}{2\Gamma_{r}^{2}}\right),$$

where $U^{*}=L^{*}/T^{*}=L^{*}\omega^{*}$ and (29) and (110) are used. The variable transformations (92) are also rewritten for consistency with Lee and Hamilton [22], [23] as

(113) $$\tau =\omega^{*}\left(t^{*}-\{1+∊[\Pi_{0}+\Pi_{4}\delta (x_{1})]\}\frac{x^{*}}{U^{*}}\right),$$

(114) $$X=\frac{x^{*}}{d_{f}^{*}}\;\left(=\frac{L^{*}}{d_{f}^{*}}\frac{x^{*}}{L^{*}}=∊x\right),$$

(115) $$R=\frac{r^{*}}{a_{r}^{*}}\;\left(=\frac{L^{*}}{a_{r}^{*}}\frac{r^{*}}{L^{*}}=\sqrt{∊}\Gamma_{r}r\right)\text{.}$$

**Fig. 4:**
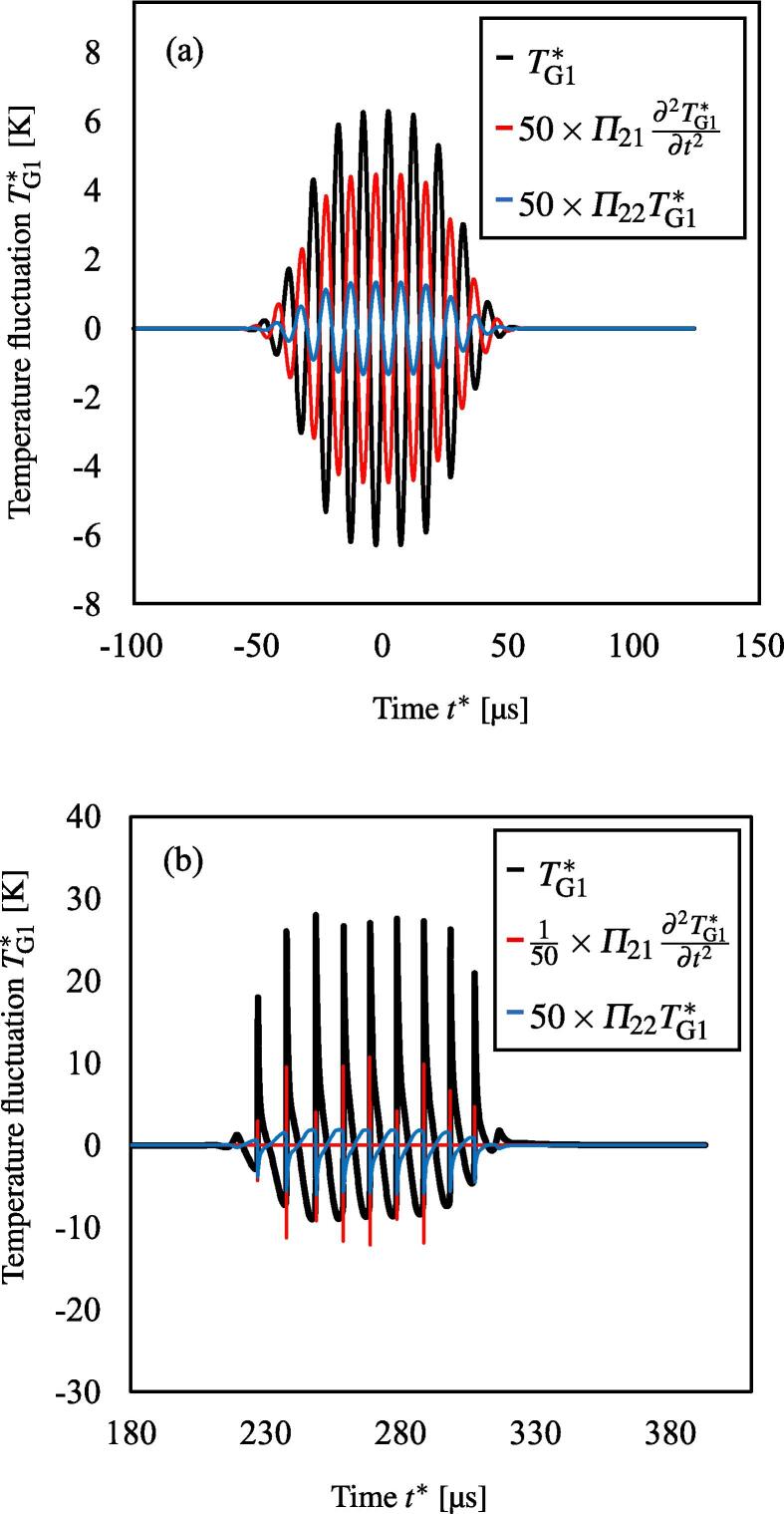
Time development of the temperature fluctuation $T_{G1}^{*}$ (a) at the sound source (boundary condition) and (b) at the focus of Fig. 3 (a), where the gas inside the bubble is Argon. The black, red, and blue curves represent the waveforms of the temperature fluctuation, the dissipation term owing to the interfacial viscosity and liquid compressibility, and the dissipation term owing to the thermal conductivity of gas.

Next, the KZK equation (111) is solved term by term to incorporate the effects of diffraction, nonlinearity, dissipation, and dispersion separately in the same manner as Lee and Hamilton [22], [23]. However, the dissipation term $\Pi_{22}T_{G1}$ and the dispersion term $\Pi_{3}\;\partial^{3}T_{G1}/\partial \tau^{3}$ in (111) are obtained by consideration of the effect of bubbles in this study. While $\Pi_{22}T_{G1}$ can be introduced easily, $\Pi_{3}\;\partial^{3}T_{G1}/\partial \tau^{3}$ is discretized for the finite difference approximation as

(116) $$\Pi_{3}\frac{\partial^{3}T_{G1}}{\partial \tau^{3}}\approx \Pi_{3}\frac{T_{G1,\;i+2}-2T_{G1,\;i+1}+2T_{G1,\;i-1}-T_{G1,\;i-2}}{2{\left(\Delta \tau \right)}^{3}},$$

where Δτ and *i* are the step size and the index in the τ direction, respectively. The boundary condition at X=0 for the focused sound source is given as

(117) $$\begin{array}{cc} & T_{G1}(\tau ,\;X=0,\;R)\\ & =\left\{\begin{array}{cc} \frac{s_{5}p_{0}^{*}}{∊s_{4}\rho_{L0}^{*}U^{*2}}E(\tau )\sin(\tau +G_{r}R^{2}) & (0\;≦\;R\;≦\;1),\\ 0 & (1<R), \end{array}\right) \end{array}$$

where $p_{0}^{*}$ is the effective pressure amplitude of the sound source, and E(τ) is the amplitude modulation function. Note that the boundary condition in the algorithm proposed by Lee and Hamilton [22], [23] was given for the liquid pressure. The example of the wave form at the sound source (i.e., the boundary condition) can be seen in Fig. 4 (a).

The step sizes for each direction are set as Δτ=2π/240, ΔX=0.001, and ΔR=0.01. In the calculations, there are three main sources of error [22], [23]; (i) finite difference approximations of the diffraction and dissipation terms, (ii) incorporating nonlinear effects based on the implicit analytical solution, (iii) including the diffraction, dissipation, and nonlinear effects separately. Considering these three main sources of error, the total error is estimated as ${\left(\Delta \tau \right)}^{2}+\Delta X+\Delta R$. Further details regarding the error are discussed by Lee and Hamilton [22], [23].

### 4.2 Result

**Fig. 3:**
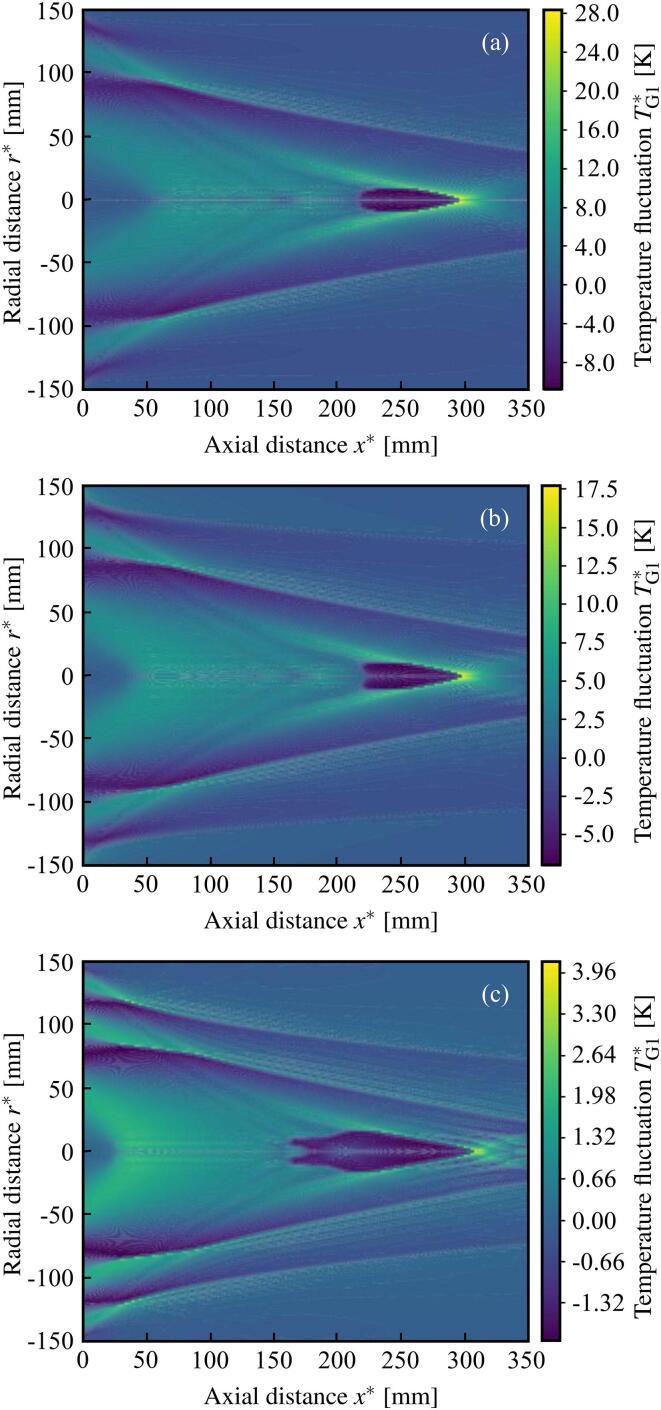
Spatial distribution of temperature fluctuations of the gas inside the bubble $T_{G1}^{*}\;[K]$, at the retarded time τ=0. The focus is on $r^{*}=0\;\mathrm{mm}$, $x^{*}=300\;\mathrm{mm}$. The types of gas inside the bubble are (a) Argon, (b) air, and (c) ${\mathrm{SF}}_{6}$.

Fig. 3 shows the spatial distribution of the first order dimensional temperature fluctuations of the gas inside the bubble $T_{G1}^{*}$, where $T_{G1}^{*}=∊T_{G1}T_{0}^{*}$ and the initial temperature $T_{0}^{*}$ is set as the normal temperature of the human body. The parameters used for calculations are; $p_{0}^{*}=100\;\mathrm{kPa}$, the frequency of the sound source $f^{*}=100\;\mathrm{kHz}$, $d_{f}^{*}=300\;\mathrm{mm}$, $a_{r}^{*}=150\;\mathrm{mm}$, $R_{0}^{*}=0.05$ μm and $\alpha_{0}=0.001$. The Shimada et al. [61] (SMK) temperature gradient model in Eq. (12) is used. In Fig. 3, the gas inside the bubble is Argon in (a), air in (b), and ${\mathrm{SF}}_{6}$ in (c). In the area near the focus at $r^{*}=0\;\mathrm{mm}$ and $x^{*}=300\;\mathrm{mm}$, an intensive temperature rise is shown. The maximum temperature rise is approximately 28.1K in (a), 17.7K in (b), and 4.13K in (c), with the Argon gas exhibiting the most effective temperature increase.

Fig. 4 shows the time development of the temperature fluctuation $T_{G1}^{*}$ at (a) $r^{*}=0\;\mathrm{mm}$, $x^{*}=0\;\mathrm{mm}$ (at the sound source) and (b) $r^{*}=0\;\mathrm{mm}$, $x^{*}=300\;\mathrm{mm}$ (focus) of Fig. 3 (a), where the gas inside the bubble is Argon. In Fig. 4 (b) at the focus, wave distortion due to nonlinear effects is shown. In Fig. 4, the time development of two types of dissipation terms; $\Pi_{21}\partial^{2}T_{G1}^{*}/\partial t^{*2}$ owing to the interfacial viscosity and liquid compressibility, $\Pi_{22}T_{G1}^{*}$ owing to the thermal conductivity of gas inside bubbles; are also shown. Although in Fig. 4 (a) at the sound source, the values of the two dissipation terms are of the same order, in Fig. 4 (b) at the focus, $\Pi_{21}\partial^{2}T_{G1}^{*}/\partial t^{*2}$ becomes dominant especially at the discontinuous point of $T_{G1}^{*}$. Note that when we use the temperature gradient models, except for the Shimada et al. [61] (SMK) model of (12), the waves rapidly attenuate even near the sound source. Hence, other temperature gradient models probably overestimate the dissipation effect of the thermal conduction of the gas inside bubbles.

Table 1 shows the values of the calculation of Fig. 3. When the gas inside the bubble is ${\mathrm{SF}}_{6}$, the value of the temperature rise and the dissipation coefficient owing to thermal conductivity $\Pi_{22}$ are the lowest of the three types. This result agrees with the analysis for the single-bubble oscillation [93]. In addition, the value of the nonlinear coefficient $\Pi_{1}/s_{5}$ is the highest. For the applications of bubbles as contrast agent for ultrasound imaging, a temperature rise is not required, but higher-harmonic generation induced by nonlinear effects is necessary. Hence, achieving a result where the temperature rise is lowest, and the nonlinear coefficient is highest for ${\mathrm{SF}}_{6}$ is consistent with the applications for ultrasound imaging.

**Table 1:** Values of the calculation of Fig. 3.

	(a) Ar	(b) Air	(c) ${\mathrm{SF}}_{6}$
Nonlinear coefficient $\Pi_{1}/s_{5}$ [–]	2.52	3.85	13.9
Dissipation coefficient $\Pi_{21}$ [–] (Viscosity and compressibility)	0.0142	0.0203	0.0351
Dissipation coefficient $\Pi_{22}$ [–] (Thermal conductivity)	-0.00427	-0.00337	-0.000193
Maximum temperature rise [K]	28.1	17.7	4.13

## 5 Conclusions

We theoretically and numerically examine a weakly nonlinear propagation of focused ultrasound in liquids containing many spherical microbubbles with a nonuniformity of bubble number density. The KZK equation [6], [7], which has long been used [8], [9], [10], [11], [12], [13], [14], [15], [16], [17], [18], [19], [20], [21], [22], [23], [24], [25], [26], [27], [28], [29], [30], [31], [32], [33], [34], [35], [36], [37], [38], [39], [40], [41] as the physico-mathematical model for focused ultrasound in pure liquids, is extended to bubbly liquids using the volumetric averaged equations for bubbly liquids based on the two-fluid model [60]. For the description of the temperature rise and the thermal effects of gas inside bubbles, the energy equation [57], (11), for the gas inside a bubble is used [55], [56], [66], [67]. The temperature gradient term in (11) is rewritten by the four major models of (12), (13), (14), (15). When we use the temperature gradient models, the numerical results show that the waves rapidly attenuate even near the sound source except for the Shimada et al. [61] (SMK) temperature gradient model of (12). Hence, it is likely that the other temperature gradient models of (13), (14), (15) overestimate the dissipation effect of the thermal conduction of the gas inside bubbles.

The resultant generalized KZK equation (90) for bubbly liquids is represented as the linear combination of nonlinear, dissipation, dispersion, and diffraction (focusing) terms. The dispersion term is owing to bubble oscillations. The diffraction terms are expressed differently for the 2D and 3D KZK equations. By using volumetric averaged equations based on a two-fluid model [60], the dependence of the nonlinear coefficient $\Pi_{1}$ (95) on the initial void fraction $\alpha_{0}$ is obtained, as shown in Fig. 2 (a). The dissipation effect is divided into the two terms; the term with the second-order derivative is due to the interfacial viscosity and liquid compressibility, and the term without differentiation is due to the thermal conductivity of the gas inside bubbles. Hence, we succeed in consistently describing ultrasound propagation, bubble oscillations, and temperature increase in a single equation.

The KZK equation is then numerically solved by the FDTD method [22], [23]. For comparison of the value of the two dissipation terms, $\Pi_{21}\partial^{2}T_{G1}^{*}/\partial t^{*2}$ being based on the interfacial viscosity and the liquid compressibility and $\Pi_{22}T_{G1}^{*}$ being based on the thermal conductivity of the gas inside the bubbles are of the same order at the sound source; however, the former becomes dominant at the focus. Finally, the spatial distribution of temperature fluctuations is obtained for Argon, air, and ${\mathrm{SF}}_{6}$ as the gas inside the bubble. As shown in Table 1, Argon gas shows the highest temperature rise, making it effective for applications in tumor ablation therapy by HIFU. ${\mathrm{SF}}_{6}$ gas exhibits the lowest temperature rise and the highest nonlinearity, making it effective for applications in ultrasound imaging.

In a future work, theoretical extensions of the KZK equation incorporating the viscous effects of bulk liquid, the elasticity of body tissues, mass transfer, heat transfer, and higher-order approximation to describe nonlinear dissipation will be carried out. Particularly, the description of blood vessels [98], [99], [100], effect of initial nonuniform distributions of velocities [101], and effect of a shell encapsulating microbubble toward ultrasound diagnosis and therapy [102] will be incorporated. Ultimately, verification of the numerical result of the extended KZK equation by comparison with experiments and direct numerical calculation is necessary.

## CRediT authorship contribution statement

**Shunsuke Kagami:** Software, Validation, Formal analysis, Investigation, Data curation, Writing – original draft, Writing – review & editing. **Tetsuya Kanagawa:** Conceptualization, Methodology, Formal analysis, Investigation, Writing – original draft, Writing – review & editing, Supervision, Project administration, Funding acquisition.

## Declaration of Competing Interest

The authors declare that they have no known competing financial interests or personal relationships that could have appeared to influence the work reported in this paper.

## Appendix A Inhomogeneous terms in (80), (81), (82), (83), (84), (85)

The inhomogeneous terms $K_{1}$ and $K_{2}$ are defined differently for the 2D and 3D KZK equations as

(A1) K1=K1^-ΓrvrG1r1/2+∂vrG1∂r1/2(for2DKZK),K1^-Γy∂vyG1∂y1/2+Γz∂vzG1∂z1/2(for3DKZK),

(A2) K2=K2^+Γr(1-α0)vrL1r1/2+∂vrL1∂r1/2(for2DKZK),K2^+(1-α0)Γy∂vyL1∂y1/2+Γz∂vzL1∂z1/2(for3DKZK),

where

(A3) K1^=-∂uG1∂x1+3∂R1∂t1-∂α1∂t1+3∂α1R1∂t0-6∂R12∂t0+3∂R1uG1∂x0-∂α1uG1∂x0+δ(x1)3∂R1∂t0-∂uG1∂x0,

(A4) K2^=(1-α0)∂uL1∂x1-α0∂α1∂t1-α0∂α1uL1∂x0+(1-α0)∂ρL1∂t0-α0δ(x1)∂uL1∂x0.

The remaining inhomogeneous terms $K_{3}$, $K_{4}$, $K_{5}$, and $K_{6}$ are common to both the 2D and 3D KZK equations

(A5) $$\begin{array}{cc} K_{3}= & 3p_{G0}\frac{\partial R_{1}}{\partial x_{1}}-p_{G0}\frac{\partial T_{G1}}{\partial x_{1}}-\beta_{1}\left(\frac{\partial u_{G1}}{\partial t_{1}}-\frac{\partial u_{L1}}{\partial t_{1}}\right)\\ & -\beta_{1}\alpha_{1}\left(\frac{\partial u_{G1}}{\partial t_{0}}-\frac{\partial u_{L1}}{\partial t_{0}}\right)-\beta_{2}(u_{G1}-u_{L1})\frac{\partial \alpha_{1}}{\partial t_{0}}\\ & +3p_{G0}\alpha_{1}\frac{\partial R_{1}}{\partial x_{0}}-6p_{G0}\frac{\partial R_{1}^{2}}{\partial x_{0}}-\beta_{1}u_{G1}\frac{\partial u_{G1}}{\partial x_{0}}\\ & +\beta_{1}u_{L1}\frac{\partial u_{L1}}{\partial x_{0}}-p_{G0}\alpha_{1}\frac{\partial T_{G1}}{\partial x_{0}}+3p_{G0}\frac{\partial R_{1}T_{G1}}{\partial x_{0}}\\ & +\delta (x_{1})\left[3p_{G0}\frac{\partial R_{1}}{\partial x_{0}}-p_{G0}\frac{\partial T_{G1}}{\partial x_{0}}-\beta_{1}\left(\frac{\partial u_{G1}}{\partial t_{0}}-\frac{\partial u_{L1}}{\partial t_{0}}\right)\right], \end{array}$$

(A6) $$\begin{array}{cc} K_{4}= & -(1-\alpha_{0})\frac{\partial p_{L1}}{\partial x_{1}}-(1-\alpha_{0})\frac{\partial u_{L1}}{\partial t_{1}}\\ & +\beta_{1}\alpha_{0}\left(\frac{\partial u_{G1}}{\partial t_{1}}-\frac{\partial u_{L1}}{\partial t_{1}}\right)+\beta_{1}\alpha_{0}\left(u_{G1}\frac{\partial u_{G1}}{\partial x_{0}}-u_{L1}\frac{\partial u_{L1}}{\partial x_{0}}\right)\\ & +\alpha_{0}\frac{\partial \alpha_{1}u_{L1}}{\partial t_{0}}+\beta_{1}\alpha_{0}\alpha_{1}\left(\frac{\partial u_{G1}}{\partial t_{0}}-\frac{\partial u_{L1}}{\partial t_{0}}\right)+\alpha_{0}\alpha_{1}\frac{\partial p_{L1}}{\partial x_{0}}\\ & +\beta_{2}\alpha_{0}(u_{G1}-u_{L1})\frac{\partial \alpha_{1}}{\partial t_{0}}-(1-\alpha_{0})\frac{\partial u_{L1}^{2}}{\partial x_{0}}\\ & -\alpha_{0}\left[-\frac{\Delta^{2}}{\Omega^{2}}R_{1}-p_{L1}+p_{G0}T_{G1}\right)\\ & \left(+3(\gamma_{e}-1)p_{G0}R_{1}+\frac{4\mu_{e0}^{2}}{\Delta^{2}}\right]\frac{\partial \alpha_{1}}{\partial x_{0}}\\ & +\delta (x_{1})\left[\alpha_{0}\frac{\partial u_{L1}}{\partial t_{0}}+\alpha_{0}\frac{\partial p_{L1}}{\partial x_{0}}+\beta_{1}\alpha_{0}\left(\frac{\partial u_{G1}}{\partial t_{0}}-\frac{\partial u_{L1}}{\partial t_{0}}\right)\right], \end{array}$$

(A7) $$\begin{array}{cc} K_{5}= & \Delta^{2}\frac{\partial^{2}R_{1}}{\partial t_{0}^{2}}+3p_{G0}R_{1}T_{G1}+\frac{4\mu_{e0}^{2}}{\Delta^{2}}R_{1}\\ & -V\Delta \left\{\left[3(\gamma_{e}-1)p_{G0}-\frac{\Delta^{2}}{\Omega^{2}}\right]\frac{\partial R_{1}}{\partial t_{0}}+p_{G0}\frac{\partial T_{G1}}{\partial t_{0}}\right\}\\ & -R_{1}^{2}\left[3(2-\gamma_{e})+\frac{\Delta^{2}}{\Omega^{2}}\right]+4\mu_{L}\frac{\partial R_{1}}{\partial t_{0}}\text{.} \end{array}$$

The explicit form of $K_{6}$ depends on which temperature gradient model is used, i.e., for (12), (13), (14), (15): [66]

(i) For (12), (14),
  (A8) K6=K6^-ζjTG1(j=SMK,LSM,PCB).
(ii) For (15),
  (A9) K6=K6^-ζSTM1TG1+ζSTM2∂TG1∂t0,

where

(A10) K6^=-3(κ-1)∂R1∂t1-∂TG1∂t1-3(κ-1)(3κ-4)R1∂R1∂t0-3(κ-1)∂TG1R1∂t0-3(κ-1)uG1∂R1∂x0-uG1∂TG1∂x0.

## Appendix B Derivation in our previous study

We briefly review the derivation in our previous study [55], [56]. The volumetric averaged conservation laws of mass and momentum based on a mixture model [58] was used:

(B1) $$\frac{\partial \rho^{*}}{\partial t^{*}}+\nabla^{*}\cdot (\rho^{*}u^{*})=0,$$

(B2) $$\frac{\partial \rho^{*}u^{*}}{\partial t^{*}}+\nabla^{*}\cdot (\rho^{*}u^{*}u^{*})+\nabla^{*}p_{L}^{*}+\nabla^{*}\cdot \tau^{*}=0,$$

where $\rho^{*}$ is the density of mixture and defined as follows:

(B3) $$\rho^{*}\equiv (1-\alpha )\rho_{L}^{*},$$

where the density of the gas phase is sufficiently smaller than that of the liquid phase, thus it is omitted. The conservation equation of the bubble number density $n^{*}$ and the relationship between the bubble number density and the void fraction are used:

(B4) $$\alpha =\frac{4}{3}\pi R^{*3}n^{*},$$

(B5) $$\frac{\partial n^{*}}{\partial t^{*}}+\nabla^{*}\cdot (n^{*}u^{*})=0\text{.}$$

The set of equations were closed by (10), (11), (12), (13), (14), (15), (16), (17), (18), (19), (20), (21), (22), (23), (24), (25). The form of the linear natural frequency of a single bubble $\omega_{B}^{*}$ was different from (16) of this paper:

(B6) $$\omega_{B}^{*}=\sqrt{\frac{3\kappa (p_{L0}^{*}+2\sigma^{*}/R_{0}^{*})-2\sigma^{*}/R_{0}^{*}}{\rho_{L0}^{*}R_{0}^{*2}}}\text{.}$$

As the same procedure of this paper, the same form of the resultant KZK equation (90) is derived. However, the coefficients of the resultant KZK equation are greatly different from this paper and given as

(B7) $${\overset{\sim }{\Pi }}_{0}=\frac{{\left(1-\alpha_{0}\right)}^{2}V^{2}}{2},$$

(B8) $${\overset{\sim }{\Pi }}_{4}=\frac{1-2\alpha_{0}}{2(1-\alpha_{0})},$$

(B9) $${\overset{\sim }{\Pi }}_{1}=1+\frac{\left(3\kappa +4)(\kappa -1\right)}{2\alpha_{0}(1-\alpha_{0})}p_{G0}+\frac{\left[3(2-\kappa )p_{G0}+\Delta^{2}/\Omega^{2}\right]}{3\alpha_{0}(1-\alpha_{0})},$$

(B10) $${\overset{\sim }{\Pi }}_{21}=-\frac{1}{6\alpha_{0}(1-\alpha_{0})}\left[4\mu_{L}\alpha_{0}(1+\alpha_{0})+4\mu_{L}+\frac{V\Delta^{3}}{\Omega^{2}}\right],$$

(B11) $${\overset{\sim }{\Pi }}_{22}=\frac{\zeta_{\mathrm{LSM}}p_{G0}(\kappa -1)}{2\alpha_{0}(1-\alpha_{0})},$$

(B12) $${\overset{\sim }{\Pi }}_{3}=-\frac{\Delta^{2}}{6\alpha_{0}(1-\alpha_{0})},$$

where the symbol ${}^{∼}$ means the coefficients of our previous study [55], [56]. Note that in our previous study [55], [56], only the 2D KZK equation was derived, and only the LSM temperature gradient model of (13) was used.
